# A Reformed British Medical Association

**Published:** 1901-02-23

**Authors:** 


### The Hospital

A Journal of

The Medical Sciences and Hospital Administration.

Vol. XXIX.—No. 752.] Edited by Sir Henry Burdett, K.C.B., and Solomon C. Smith, M.D., M.R.C.P. [February 23, 1901.

### A Reformed British Medical Association.

The British Medical Association has become so large a body, and with increasing numbers its potentialities for good or evil are so considerable, that any alteration in its constitution is a matter of interest to all practitioners of medicine, whether members of the Association or not.

Those who are cognisant of the almost riotous proceedings which for years past have characterised many of the general meetings of the Association will perhaps be surprised to find how simple a matter reform now appears to be. For years back the angry passions of a crowd of private members have beaten against the impassive front presented by the Council without avail. Just a little word was wanted—a little word which, coming in the form of a "legal opinion," to the effect that the Council was bound to do as it was told, made all the difference. Reformers fuss and fume and think they do great deeds. But reform comes when the time for it is ripe; and in this case the time became ripe at once when counsel spoke that little word and said that the annual meetings, with all their umbrella thumpings and other objectionable habits, must be obeyed. So a committee was appointed to sketch out a new constitution, and now it has presented its report.

According to the scheme put forward in this report, the Council will be made smaller and more directly representative of the branches, but it will remain the executive body, and will retain the entire management of the affairs of the Association. Moreover, although it must still do as it is bid by the general meeting, it can only be so bid under certain limitations, and if it considers its instructions injurious to the interests of the Association it can refer the matter to the members, a form of referendum being arranged by which the opinion of the whole body of members can be obtained.

The great and striking change, however, which is proposed is that instead of the primary unit of local organisation being the Branch it shall be a much smaller body, to be called the Division, each Division consisting, as far as possible, of such members as reside within the area of a parliamentary constituency. The distinction between attached and unattached members is to be done away with. Every member resident in the United Kingdom becomes *ipso facto* a member of the Division in which he resides, and, of course, of the Branch of which his Division forms a part. He will, as at present, be able to vote at the general business meetings of the Association, but some limits will be placed upon the subjects which can there be lawfully brought forward; and as the quorum will be raised to two hundred and a majority of two-thirds will be required for anything touching the funds, and as the Council will always be able to appeal by referendum, the irresponsible private member will find his wings somewhat clipped.

The position, however, of the private member who voices a topic congenial to even a few of his fellows will be much improved. As before, he will be a member of his Branch, on the deliberations of which he can exercise such influence as he possesses. But he will also be a member of his Division, a much smaller body on which any man who chooses so to do may certainly make himself a person of some importance; and as it is arranged that every Division shall send its own delegate, at the expense of the Association, to attend the committee of delegates at the annual meeting; that resolutions "relating to the maintenance of the honour and interests of the medical profession" may after certain formalities be brought before this committee by any Division; and that such resolutions if affirmed by two-thirds of the votes given shall be regarded as the decision of the Association, and shall be binding on the Council, it is clear that the private member will have far more influence in the affairs of the Association than can at present be obtained by any except the noisy ones. The suggested reform is all in favour of the steady-going members, and tends to diminish the evil preponderance of the orators. The arrangments for representation and the restrictions proposed by the constitution committee are very elaborate and far too complicated to be discussed here. On the whole they seem well calculated to fulfil their purpose, to maintain the stability of the Association, to extend the representative character of its government, and very much to increase the power of individual members to initiate movements relating to the honour and interests of the profession. But outside and beyond the bearing of the proposed changes on the conduct of the affairs of the Association we think that the proposed reforms will have a very widespread influence for good upon the medical profession at large. Practically the new constitution means the establishment of a medical society, little or big, in every parliamentary division throughout the United Kingdom, and this alone will be an enormous gain to the whole profession.

The Branch meetings have been almost everywhere a failure: the Branches are too large, the distances are too great, the holiday making has been too obvious to tempt many members to attend them; but with a medical society in every town the matter would be very different. Such a medical society would not be a shifting body, growing or shrivelling according to the energy or idleness of its secretary, but would consist of every resident member of the Association. It would be a permanent nucleus; a recognised body sending its own delegate to the annual meeting, able to initiate reform, and able to ensure that its opinion shall be brought before the whole body of delegates in general meeting assembled. The establishment of such societies all over the United Kingdom ought to have an untold influence for good in encouraging intercourse and friendly feeling among their members, in promoting solidarity and unity of action, and in giving mutual help to those who do good work in their profession. That such a scheme will in the end greatly increase the number of members of the Association we can hardly doubt.

